# Polygenic discrimination of migratory phenotypes in an estuarine forage fish

**DOI:** 10.1093/g3journal/jkac133

**Published:** 2022-05-30

**Authors:** Matthew A Campbell, Shannon E K Joslin, Alisha M Goodbla, Malte Willmes, James A Hobbs, Levi S Lewis, Amanda J Finger

**Affiliations:** Genomic Variation Laboratory, Department of Animal Science, University of California, Davis, Davis, CA 95616, USA; Genomic Variation Laboratory, Department of Animal Science, University of California, Davis, Davis, CA 95616, USA; Genomic Variation Laboratory, Department of Animal Science, University of California, Davis, Davis, CA 95616, USA; Institute of Marine Sciences, UC Santa Cruz, Santa Cruz, CA 95064, USA; National Marine Fisheries Service, Southwest Fisheries Science Center, Santa Cruz, CA 95064, USA; Otolith Geochemistry and Fish Ecology Lab, Department of Wildlife, Fish and Conservation Biology, University of California, Davis, Davis, CA 95616, USA; Otolith Geochemistry and Fish Ecology Lab, Department of Wildlife, Fish and Conservation Biology, University of California, Davis, Davis, CA 95616, USA; Genomic Variation Laboratory, Department of Animal Science, University of California, Davis, Davis, CA 95616, USA

**Keywords:** adaptive genetic variation, Delta Smelt, migration, Osmeridae, resident ecotype, semianadromy

## Abstract

Migration is a complex phenotypic trait with some species containing migratory and nonmigratory individuals. Such life history variation may be attributed in part to plasticity, epigenetics, or genetics. Although considered semianadromous, recent studies using otolith geochemistry have revealed life history variation within the critically endangered Delta Smelt. Broadly categorizable as migratory or freshwater residents, we examined Restriction site Associated DNA sequencing data to test for a relationship between genetic variation and migratory behaviors. As previously shown, we found no evidence for neutral population genetic structure within Delta Smelt; however, we found significant evidence for associations between genetic variants and life history phenotypes. Furthermore, discriminant analysis of principal components, hierarchical clustering, and machine learning resulted in accurate assignment of fish into the freshwater resident or migratory classes based on their genotypes. These results suggest the presence of adaptive genetic variants relating to life history variation within a panmictic population. Mechanisms that may lead to this observation are genotype dependent habitat choice and spatially variable selection, both of which could operate each generation and are not exclusive. Given that the population of cultured Delta Smelt are being used as a refugial population for conservation, as a supply for wild population supplementation, and currently represent the majority of all living individuals of this species, we recommend that the hatchery management strategy consider the frequencies of life history-associated alleles and how to maintain this important aspect of Delta Smelt biological variation while under captive propagation.

## Introduction

The causes of migration may be viewed as both proximate and ultimate (Tinbergen 1963). Ultimate causes are concerned with the broader evolutionary mechanisms, origins, and consequences of migration. The scale of these questions allows comparison across taxa and unifying themes to be proposed such as the increasing food availability hypothesis and diadromy in fishes (Gross *et al.* 1988). Proximate causes are focused on individuals and the expression of migratory (MIG) behavior and associated traits in response to environmental cues, genetic background, and nongenetic parental affects (e.g. Ferguson *et al.* 2019). These proximate questions are much more limited in the applicability of results across taxonomic levels. In particular, genetic contributions to the control of migration have been widely documented across the literature (e.g. Liedvogel *et al.* 2011); however, the identification of a shared genomic region associated with MIG timing in 2 divergent species of Pacific salmon (*Oncorhynchus*) is atypical (Prince *et al.* 2017).

Studying the causes of migration relies on the characterization of MIG syndromes—a compendium of movement patterns, behavioral, morphological, and physical traits—of individuals within a population (Sih *et al.* 2004; Dingle 2006). In the most extreme cases, such as partial migration, individuals within the same population exhibit different behaviors, with some individuals (migrants) making movements across habitats, and other nonmigrating individuals (residents) completing their life cycle within a single habitat type (Chapman *et al.* 2012; Kendall *et al.* 2015; Hobbs *et al.* 2019). Individuals of a species exhibiting different MIG syndromes are subject to different selection pressures in the form of suites of predators, physical environmental conditions, trophic resources, growth rates, and anthropogenic disturbances (Gross 1987; Moyle and Cech 2004). The complexity of alternative MIG syndromes may be reflected by a complex genetic background with hundreds of loci covarying between distinct ecotypes (e.g. Pavey *et al.* 2015). Life history variation and ecotypic differentiation may also be influenced by multiple genes (e.g. over 1,000) that are consolidated into supergenes (Thorneycroft 1975; Lowry and Willis 2010; Pearse et al. 2014, 2019; Tuttle *et al.* 2016), with the possibility of multiple (2 to several) supergene complexes present within a single species (Huang *et al.* 2020; Campbell *et al.* 2021). Despite this potential complexity, large differences in behavioral phenotypes result from minor differences in the genome. For example, major differences in migration timing (e.g. spring-run vs fall-run Chinook Salmon) are associated with a simple Mendelian polymorphism (Thompson *et al.* 2020). Epigenetic control of migration in Rainbow Trout (*Oncorhynchus mykiss*) has been demonstrated (Baerwald *et al.* 2016), indicating that heritable nongenetic variation can be considered. In contrast, it is also possible that life history and ecotypic diversity may simply represent phenotypic plasticity without a heritable foundation (Gotthard and Nylin 1995).

Although California’s critically endangered Delta Smelt (*Hypomesus transpacificus*) has been broadly described as semianadromous (Moyle *et al.* 1992; Sommer *et al.* 2011), recent results based on otolith geochemical analyses have identified complex MIG histories in this species (Hobbs *et al.* 2019). Otoliths are calcium carbonate structures in the inner ear of most bony fishes that form continuously throughout the life of a fish (Campana 1999). The geochemical analysis of the otoliths can be used to reconstruct movement between different habitats, along rivers and within the different salinities of estuarine environments. As originally described, the Delta Smelt population is dominated in most years by a semianadromous MIG phenotype that spawns in freshwater and rears in brackish waters of the San Francisco Estuary. However, Hobbs *et al.* (2019) also identified a significant fraction of the population as freshwater residents (FWR) as well as a rarer phenotype that hatches and rears in low-salinity brackish habitats (BWR). Although Delta Smelt can be found in different regions of the estuary, no geographic population structuring has been identified for this species, thus it is managed as a single panmictic population (Fisch *et al.* 2011).

Given that the Delta Smelt population is now <1% of its former levels (Moyle *et al.* 2016; Hobbs *et al.* 2017), the survival of the species now rests largely on hatchery production, which serves as both a genetic-reserve population and a source of fish for supplementation of the wild population. Although culture practices have attempted to follow sound genetic management guidelines, this recently has been hampered by limited introductions of genetic diversity coming from the few wild-caught broodstock in each year. Due to the decline of the naturally reproducing Delta Smelt no natural origin fish contributed to hatchery broodstock in 2020 and 2021. Furthermore, strong hatchery domestication pressures exist on the reserve population (Finger *et al.* 2018). Any potential genetic diversity associated with life history variation has yet to be considered in the genetic management of the species, thus leaving captive propagation and planned genetic monitoring following the release of cultured individuals into the wild uninformed with respect to life history diversity.

Here, we conduct an interdisciplinary study, combining the results of otolith geochemistry and Restriction site Associated DNA sequencing (RADseq), to investigate several important questions regarding the potential genetic underpinning for the MIG behaviors of Delta Smelt and related implications for species management and conservation. Specifically, we examine whether there is evidence for a genetic basis of life-history variation within Delta Smelt, how any such genetic basis might be characterized, and how well the MIG phenotype of an individual can be predicted from its genotype. Results of this work illuminate the control of MIG behaviors in Delta Smelt and how such phenotypic diversity might be incorporated into culture and supplementation strategies.

## Materials and methods

### Samples and phenotypic classification

The Delta Smelt were collected by the California Department of Fish & Wildlife’s Spring Kodiak Trawl Survey between January and March 2013. Each fish was measured fresh in the field, given a unique individual serial number, and archived in liquid nitrogen. Otoliths were extracted and prepared for geochemical analysis following methods detailed in Hobbs *et al.* (2019). Otoliths in this study were analyzed in situ at the UC Davis Interdisciplinary Center for Plasma Mass Spectrometry using a multicollector inductively coupled plasma mass spectrometer (*Nu Plasma HR* from Nu Instrument Inc.) interfaced with a Nd:YAG 213 nm laser (New Wave Research UP213) (LA–MC–ICP–MS). Strontium isotope ratios (^87^Sr/^86^Sr) were obtained from the core to the ventral edge of the otolith, representing the entire lifespan of the fish. Individuals expressing an FWR phenotype exhibit ^87^Sr/^86^Sr profiles with values remaining below 0.7075 across the entire profile (entire life span), corresponding with practical salinities <0.5 (i.e. “freshwater”) (Hobbs *et al.* 2019). In contrast, individuals expressing a semianadromous MIG phenotype exhibit ^87^Sr/^86^Sr profiles that begin at values <0.7075 for at least the first 30 days posthatch, followed by a transition into brackish-water habitats, identified by ^87^Sr/^86^Sr values between 0.7075 and 0.7092.

### Genetic analysis

Total genomic DNA was extracted from tissues using a Qiagen DNeasy extraction kit following the manufacturer’s protocols. RADseq libraries were generated with the *SbfI* enzyme following the Best Rad protocol (Ali *et al.* 2016). Libraries were sequenced with 100 base pair paired-end sequencing on an Illumina HiSeq 4000 at the UC Davis Genome Center.

Sequence data files were aligned to the Delta Smelt reference genome (GCA 021917145.1) with the Burrows–Wheeler Aligner using the MEM algorithm (Li and Durbin 2009). Resulting alignments were sorted, PCR duplicates removed and coverage in terms of the number of aligned reads were calculated with SAMtools (Li *et al.* 2009). We removed individuals from the analysis that constituted the bottom 25% of coverage in terms of aligned reads and lacked complete phenotype classification and sex metadata. For all analyses, we analyzed assembled contigs of the Delta Smelt genome >500 kb totaling 400,418,546 bases representing 92% of bases in the assembly. The contigs larger than 500 kb in the assembly numbered 73 with a total 376 contigs in the assembly.

We searched for a signal of neutral genetic structure within sampled Delta Smelt with PCAngsd through principal component (PC) analysis (Meisner and Albrechtsen 2018). PCAngsd required a genotype likelihood file that we generated through ANGSD using a SAMtools likelihood model (-GL 1) in the Beagle format (-doGlf 2) (Korneliussen *et al.* 2014). For quality control thresholds, we required a site to be present in 90% of individuals (-minInd 108), a significance value of 1e−6 (-SNP_pval), a minimum mapping quality of 20 (-minMapQ), and a minimum base quality of 20 (-minQ). The resulting genotype likelihoods were analyzed with the default settings of PCAngsd, which includes a minimum minor allele frequency (MAF) of 0.05.

Genetic variants associated with life history variation were identified through a genome-wide association study (GWAS) implemented in ANGSD. We used a generalized linear framework (-doAsso 2) and provided sex as a covariate (-cov). We applied the following options to the program to provide quality control -minMapQ 20, -minQ 20, -SNP_pval 1e6, and -minInd 91. We specified a SAMtools genotyping model (-GL 1) and -minCount 2. To determine a significance threshold, we do not use a Bonferroni correction as sites in the genome are not independent. Instead, we calculated a significance threshold in a Bayesian framework (e.g. Burton *et al.* 2007). We assumed for the calculation of a prior probability that life history variation in Delta Smelt is polygenic and that closely linked variants to SNPs underlying phenotypic differences would also be detected by GWAS. Following this assumption, we assumed for calculation of a prior probability, *P*(*T*), that there are 25 genetic variants that may contribute to life history variation within the GWAS, *P*(*T*) = 25/number of variants. The significance level can be calculated by the formula: α = Prior Odds * 1/Posterior Odds (e.g. Wacholder *et al.* 2004), where prior odds is *P*(*T*)/(*1−* *P*(*T*)), posterior odds is calculated based on 95% certainty of observing a significant effect that is real (0.95/(1*−* 0.95)) and an upper bound of power (1) is used.

We examined the strength of the signal in the genetic data to separate Delta Smelt in FWR and MIG categories with discriminant analysis of PCs (DAPC), hierarchical clustering, and machine learning approaches. With these approaches we used called genotypes generated by ANGSD (-doGeno 2) producing a dataset of 0, 1, 2 coded variation. We applied the same quality control thresholds as used previously, with the following changes: an MAF specified (-minMaf 0.05); posterior cutoff specified (-postCutoff 0.9), and a 90% missing individual threshold (-minInd 109). The genotypes were uploaded into R for further analysis (R Development Core Team 2020).

DAPC identifies the largest axis of variation between predefined groups. We conducted DAPC using the adegenet package in R (Jombart 2008) with MIG phenotype supplied as an a priori grouping variable. We visualized the ability of DAPC to classify the fish into phenotype classes by visualizing the first discriminant function and generating histograms of the posterior probability of assignment to prior classes. We also examined the contributions (loadings) of each site to the separation of phenotypic classes. Separately we investigated the predictive power of genotypes to predict phenotype. We examined the top 200 associated variants identified by GWAS with ANGSD for respective genotype calls and then generated a heatmap with the *heatmap.2* function of the gplots package. Clustering of samples and loci was done with Ward’s distance, and missing data were not treated. The same set of 200 most-associated SNPs was then analyzed with a *k*-nearest neighbor (knn) approach for classification after treating missing data with the *na.roughfix* function of the randomForest package. We divided our dataset into a training dataset of 30 randomly selected FWR and 30 randomly selected MIG fish. With the training dataset, we identified a best *k* with a repeated *k*-fold cross validation using the *trainControl* and *train* functions of the caret library. A best *k* was identified as being most accurate after 100 sampling events and 10-folds for odd values of *k* from 1 to 29. The selected best *k* was then applied to all samples for the top associated variants and a cross table of accuracy computed with the *CrossTable* function of the gmodels library.

### Functional analysis

Annotated genes located near SNPs distinguished by GWAS were identified to provide insight into the functional relevance of the polymorphisms to MIG phenotypes. We determined first the extent to which linkage disequilibrium (LD) operates in the Delta Smelt genome given our data, and then obtained annotated features within that window. Descriptions of genes, Gene Ontology (GO) terms, GO names, and GO definitions were obtained with the biomaRt package in R by querying the zebrafish dataset (drerio_gene_ensembl) (Durinck *et al.* 2005, 2009).

## Results

**Fig. 1.**
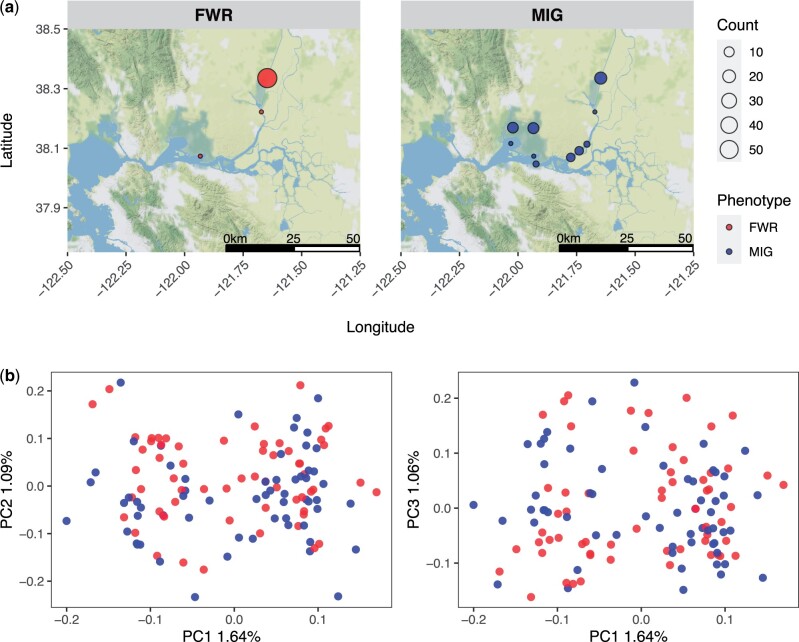
a) The geographic distribution of Delta Smelt samples examined in this study, facets are split between FWR and MIG individuals. b) A PC analysis of individuals examined in this study. The facets are split between a plot of PC1 vs PC2 and PC1 vs PC3.

**Fig. 2.**
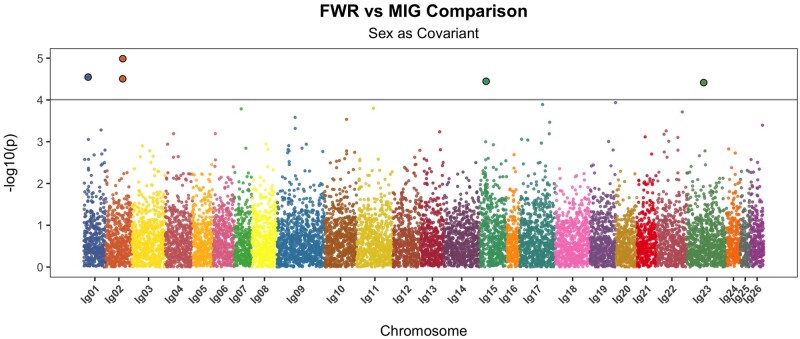
Manhattan plot of genome-wide association testing contrasting FWR and MIG Delta Smelt individuals. Sex was provided as a covariant and the linkage groups of the Delta Smelt genome assembly are shown. Sites exceeding the significance threshold are indicated with larger filled circles and the significance level [*P *=* *9.9e−05, −log10(*P*) = 4.00].

After filtering for coverage and complete metadata, our dataset contained 60 FWR and 61 MIG individuals closely split between sexes (Table 1). Sample metadata is reported in Supplementary Document 1 with FWR individuals largely restricted to the northern region of the Sacramento-San Joaquin Delta while MIG individuals were more-widely distributed (Fig. 1a). Based on the reference assembly, a theoretical number of RAD loci based on the *SbfI* restriction site is 16,578. Genotype likelihoods were identified from 11,638 RAD loci (63%, Supplementary Appendix 1). PC analysis of 18,765 SNPs did not suggest that phenotype classification and genetic structuring were closely related overall (Fig. 1b). We identified 5 significantly associated variants from 13,376 sites examined by GWAS located on 4 linkage groups (Fig. 2 and Table 2). Two of the most-associated variants were found in close proximity, only 153 bp apart, on lg02. The complete association test results are provided as Supplementary Document 2.

**Table 1.** Summary of Delta Smelt examined in this study reported by phenotype and sex.

Phenotype	Sex	Average aligned reads	Sample size	Total *N* for phenotype
FWR	Male	1,080,559	34	60
FWR	Female	1,022,383	26	
MIG	Male	892,302	32	61
MIG	Female	1,091,578	29	

**Table 2.** Most highly associated genetic variants from association testing.

Chromosome	Position	Major	Minor	Frequency	*P-*value
lg01	2467271	G	A	0.09	2.8 × 10^−5^
lg02	11230311	A	C	0.31	3.1 × 10^−5^
lg02	11230464	T	G	0.30	1.0 × 10^−5^
lg15	2268817	C	T	0.24	3.6 × 10^−5^
lg23	7996307	G	A	0.15	3.9 × 10^−5^

For each site the chromosome, position, major allele, minor allele, minor allele frequency, and *P-*values are reported.

**Fig. 3.**
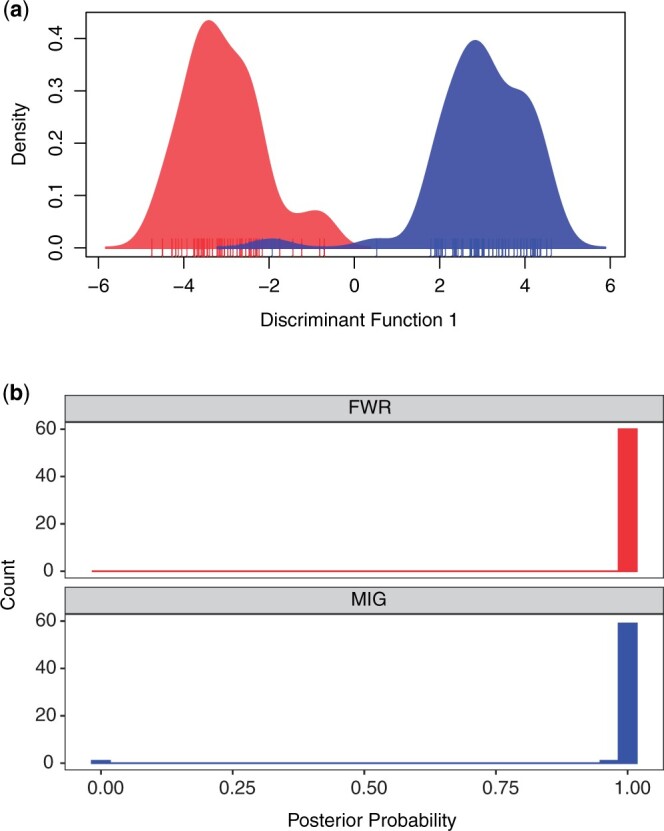
a) Density of Delta Smelt individuals along the discriminant function generated by DAPC. Red indicates FWR and blue MIG. Individuals are indicated with a carpet plot. b) The posterior probability of fish being assigned to the phenotypic class of origin. FWR individuals are plotted in a facet in red and MIG are plotted in a second facet in blue.

Calling genotypes produced 9,068 variants (provided as an R data binary as Supplementary File 1) composed of 7,274 independent SNPs that do not exhibit genetic structuring between FWR and MIG fish and represent ∼11,090 RAD loci (Supplementary Appendix 1). Within DAPC, we specified 110 PCs (n.pca = 110) and a single axis for discriminant analysis (n.da = 1). The samples largely were separated into 2 groups with high posterior probability of assignment (Fig. 3). FWR individuals had a mean posterior assignment probability of 0.999 to the FWR class and 6.77 × 10^−4^ to the MIG class. The mean posterior assignment for the MIG to MIG class was 0.983, with 1.70 × 10^−2^ of MIG fish to the FWR class. A single MIG individual had a FWR posterior assignment of 1.00, causing a large overall reduction in MIG posterior assignment probability. The sites contributing more than 0.0005 to the loadings are provided in Supplementary Document 3.

**Fig. 4.**
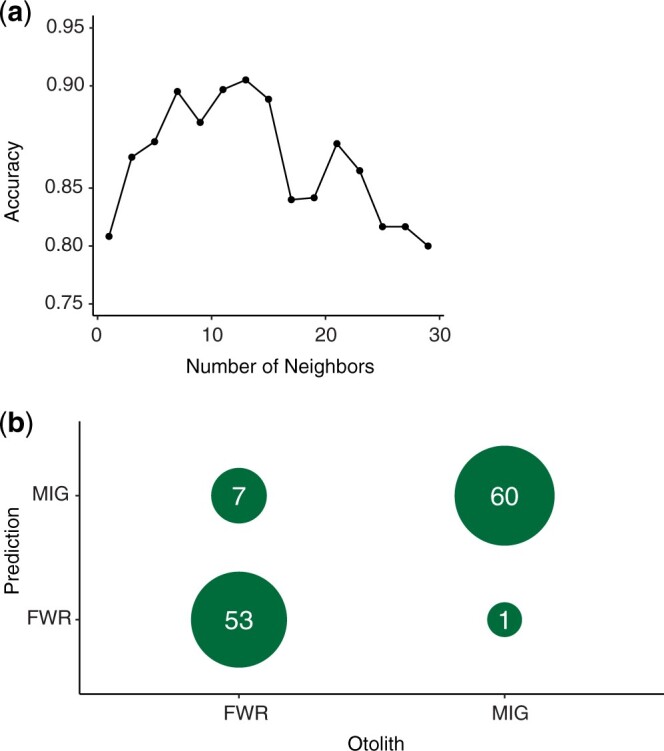
Results of *k*-nearest neighbor classification of life history phenotypes. a) Overall accuracy from cross-validation identifying an optimal number of neighbors with half the dataset as a training set (0.94, *k *=* *13). b) The success in assigning the dataset to categories based on the training dataset as a cross table.

Hierarchical clustering with the 200 genotypes having the strongest phenotypic association creates 2 major groupings each representing a majority FWR or a majority MIG (Supplementary Fig. 1). Most, 88% (53/60), of FWR compose a cluster and most, 95% (58/61), of MIG individuals compose the other cluster. The same 200 genotypes exhibited the highest accuracy with knn with *k *=* *13 neighbors (94%, Fig. 4a). Subsequently, we were able to successfully assign 88% of FWR individuals and 98% of the MIG individuals for an overall accuracy of 94% (Fig. 4b).

For obtaining genes near the 5 SNPs identified by GWAS, we determined that LD in Delta Smelt did not extend widely in the genome (Supplementary Document 4). A conservative 26,500 bp range was searched around identified SNPs, except in the case of the 2 SNPs occurring in close proximity on lg02, where a single region was searched but extended by the distance between those 2 SNPs. Nine genes are annotated and named nearby to highly associated SNPs (Table 3). GO names and descriptions are reported in Supplementary Document 4.

**Table 3.** Annotated genes near (within 26,500 base pairs) of most highly associated genetic variants from association testing.

Chromosome	Feature start	Feature stop	Strand	Gene symbol	Gene description
lg01	2480847	2497813	−	*nfkb1*	Nuclear factor of kappa light polypeptide gene enhancer in B-cells 1
lg02	11219956	11224590	+	*nrsn1*	Neurensin 1
lg02	11243757	11253748	−	*slc6a3*	Solute carrier family 6 member3
lg15	2261150	2266523	+	*aamdc*	Adipogenesis associated, Mth938 domain containing
lg15	2266390	2274233	−	*ints4*	Integrator complex subunit 4
lg15	2274295	2276264	−	*kctd14*	Potassium channel tetramerization domain containing 14
lg15	2276521	2279995	−	*rps3*	Ribosomal protein S3
lg23	7979005	7986497	−	*tfcp2l1*	Transcription factor CP2-like 1
lg23	7987047	8045995	−	*clasp1a*	Cytoplasmic linker associated protein 1a

The chromosome, feature start, feature stop, strand, gene symbol, and gene description are given.

## Discussion

### Summary of results/conclusions

In this study we examined genotype–phenotype associations with respect to the life history of Delta Smelt by combining otolith Sr isotope geochemistry and genetic sequencing. Delta Smelt exhibit a complex life history known as partial migration where some portion of the population spawn in freshwaters of the Sacramento-San Joaquin Delta and disperse downstream to rear in estuarine habitats within the SFE before returning to spawn in freshwater (semianadromous migrants, MIG); while others complete their life cycle entirely within freshwater habitats, foregoing migration (FWR) (Hobbs *et al.* 2019). The presence of a genetic foundation for this observed variation in MIG behaviors and adaptive genetic variation relevant to these alternative selective regimes is an important gap in our understanding of the molecular mechanisms underlying life history diversity in this species. Overall, we found no apparent population-level genetic structure in terms of genome-wide nucleotide diversity as has been previously reported (Fisch *et al.* 2011) (Supplementary Appendix 1), however, we discovered a significant association between phenotypic and genotypic variation, suggesting that life history complexity in Delta Smelt exhibits a heritable genetic foundation, indicative of evolutionary processes that select for diverse traits. Outwardly, the MIG fish were larger than the FWR as measured through fork length, which is in agreement with the increased foraging success of MIG fish (Hammock *et al*. 2017). Though more work is needed, translation of this adaptive genetic variation into management-relevant tools remains an under-explored and likely valuable option for improving conservation and recovery efforts for this imperiled endemic species.

### Evolutionary origin of Delta Smelt and Delta Smelt life history diversity

Although the observed life history diversity in Delta Smelt has likely existed for a long time, it remains unclear whether semianadromy has always been favored, or if it is only currently dominant due to recent natural or anthropogenic forcings. The evolutionary origins of Delta Smelt point to a mid-Pliocene to early Pleistocene divergence from Surf Smelt (*H. pretiosus*), a widely distributed marine species (Ilves and Taylor 2007). A likely scenario is that Delta Smelt diverged through glacial isolation in a freshwater basin in western California, such as in the Pleistocene lakes of the southern San Joaquin Valley (Norris and Webb 1990). Thus, historically, freshwater inputs from rain, springs, and snowmelt likely provided large expanses of cool freshwater habitats for FWR Delta Smelt, and postglaciation, Delta Smelt were reconnected with the estuary and likely thrived in both freshwater and estuarine habitats.

However, warming temperatures, hydrological alterations, and habitat destruction have dramatically altered Delta Smelt habitats [Cloern *et al.* 2011; San Francisco Estuary Institute-Aquatic Science Center (SFEI-ASC) 2014; Hobbs *et al.* 2017; Hutton *et al.* 2017]. Contemporary FWR Delta Smelt are now largely relegated to the Sacramento Deep Water Ship Channel in the northern region of the Sacramento-San Joaquin Delta and are abundant only in the coolest of years; whereas MIG Delta Smelt are found more widely throughout the SFE and are more abundant in years with average and above average temperatures (Hobbs *et al.* 2019; Lewis *et al.* 2022). The complex life history of Delta Smelt is likely a result of pre-existing adaptation to a wide variety of habitats historically available in the Sacramento-San Joaquin Delta (Hobbs *et al.* 2019), and its ancient origin constitutes an important aspect of the species’ biology that is critical for informing the long-term management of the species.

### Genetic association vs causative polymorphisms

Species may respond to heterogeneous environments through local adaptation or phenotypic plasticity. Evidence for population genetic structure and associated neutral genetic divergence indicating local adaptation is lacking in Delta Smelt (Fig. 1b). The alternative view, that phenotypic plasticity is underlying life history variation in Delta Smelt is undercut by our results indicating that there is a genetic association with MIG phenotypes in Delta Smelt that is polygenic (Fig. 2). Two nonexclusive hypotheses may be proposed to explain the observed patterns in Delta Smelt: genotype-dependent habitat choice, and intragenerational spatially varying selection that results in ecotypic differentiation but not large genetic differentiation (e.g. Pavey *et al.* 2015). These 2 hypotheses would recreate generationally the observed patterns within Delta Smelt in terms of phenotype and genotype and have been observed in other organisms (e.g. Bourret *et al.* 2014; Soria-Carrasco *et al.* 2014; Pavey *et al.* 2015).

The reduced representation sequence data used in this study are unlikely to sample the causative polymorphisms observed but should be able to sample regions that are in LD with a causative genome region. As a result, the variants identified are likely not causative polymorphisms, but may exhibit some linkage to causative polymorphisms. Evaluation of the functional differences reflected by observed genotypic differences rests on further evaluation with more complete genome coverage and understanding of the Delta Smelt’s genome. Very strong associations and extremely high assignment accuracy are not expected given the data type; however, we did identify statistically significant associations with life history variation and had high classification success. Overall, we did not identify a clear single region of association; rather at least 4 separate chromosomal regions were implicated by GWAS. The strongest association identified on lg02 (site 11230464) also contributed the greatest to the separation of FWR and MIG fish through DAPC (Supplementary Document 3). As a whole, the genetic signatures of FWR and MIG fish permitted classification with >90% accuracy into phenotypic class with simple algorithms (Ward’s distance clustering, knn). Several fish were not categorized correctly (7 FWR individuals and 1 MIG). All of these fish came from the same sampling location where most FWR were sampled (Supplementary Appendix 1). The more geographically dispersed samples of FWR fish; however, were assigned correctly.

### Candidate genes for MIG phenotypes

The functional analysis conducted did identify several genes that may relate to the observed phenotypes. The mostly high-associated SNP on lg02 is located near solute carrier family 6 member 3 (*scl6a3*), which functions in transmembrane sodium transport (GO: 0035725, Supplementary Document 4). Members of the solute carrier family 6 including *scl6a3* have been previously associated with adaptation to salinity variation in threespine sticklebacks (*Gasterosteus aculeatus*) as well as a *scl6a3* specifically being identified as a candidate gene from freshwater and marine divergence (Hohenlohe *et al.* 2010; Guo *et al.* 2015).

The transcription factor nuclear factor of kappa light polypeptide gene enhancer in B-cells 1 (*nfkb1*) is located near a highly associated SNP and is a key regulator of immune response (Oeckinghaus and Ghosh 2009). MIG fishes inhabit different environments and thus are exposed to alternative parasites and pathogens in those alternative habitats. Furthermore, immunity is an energy intensive process and may result in trade-offs with other energy intensive processes such as fecundity and movement (Colgan *et al.* 2021). Between FWR and MIG phenotypes, immune-related genes may be under strong selective processes. A second transcription factor was also identified in our functional analysis: transcription factor CP2-like 1 (*tfcp2l1*). This transcription factor is not well-characterized in fishes, however, the mouse ortholog is known to function in the development of kidneys and the diversification of both intercalated and principal cells regulating acid–base and salt–water homeostasis (Werth *et al.* 2017). Salinity levels and thus requirements for osmoregulation are distinctly different within and between the life cycles of FWR and MIG Delta Smelt.

The adipogenesis associated, Mth938 domain containing gene (*aamdc)* was not assigned GO terms in ENSEMBL, but the UniProt database indicates a function of positive regulation of fat cell differentiation (https://www.uniprot.org/uniprot/Q502H1). Adiposity is previously been linked to MIG behavior in fishes. Examples include vestigial like family member 3 (*vgll3)* which is an adiposity regulator that exhibits a high degree of control of age-at-maturity in Atlantic Salmon (*Salmo salar*) (Barson *et al.* 2015), and genes linked to adiposity (*rorc1*, *rxra*, and *lepr*) are found in the key chromosomal inversion underlying divergent MIG phenotypes in Rainbow Trout (Pearse *et al.* 2019).

### Management implications

The Sacramento-San Joaquin Delta is now unable to sustain large numbers of Delta Smelt, with captive Delta Smelt comprising nearly all existing members of the species (Lessard *et al.* 2018). As our results indicate both FWR and MIG genetic backgrounds in this population, the preservation of life history-associated variants during captive propagation is needed in order to maintain the full portfolio of Delta Smelt life histories in the cultured population. Many diadromous species have evolved diverse portfolios of MIG behaviors that allow them to persist within stochastic environments. Partial migration (co-occurring MIG and resident phenotypes), for example, can enhance the stability and resilience of populations to natural and anthropogenic disturbances (Lundberg 1988; Greene *et al.* 2010) by serving as a bet-hedging strategy that spreads risk and enhances resilience to environmental stochasticity (Roff 1993; Rochet 2000; Schindler *et al.* 2010; Moore *et al.* 2014; Hodge *et al.* 2016; Brennan *et al.* 2019). Once quantified, this intraspecific phenotypic variation in MIG behaviors can be translated into management-relevant tools that are key to effective management and conservation (Schindler *et al.* 2010). Management practices should account for and incorporate such variation, as focusing on a single life history phenotype may not maximize population stability in the long-term (Hilborn *et al.* 2003; Greene *et al.* 2010; Schindler *et al.* 2010; Brennan *et al.* 2019).

In addition to monitoring life history associated variants in the hatchery population, continued monitoring of highly associated variants through the course of the reintroduction effort is also advisable. Although the genetic variants identified in this paper are useful for these purposes, there are limitations in genome coverage and the Delta Smelt in this study are all from a single brood year (2012). Monitoring of Delta Smelt life history adaptive genetic variation would be best served through the identification of genetic or structural variants more closely linked to life history variation through whole-genome sequencing and corroboration across more brood years of Delta Smelt.

### Future research

Though informative, here we only examined a single cohort of Delta Smelt. The importance and prevalence of these associations through time are unknown and genome coverage was not complete. Furthermore, patterns in the relative abundance of each phenotype are likely to covary with changes in environmental conditions that may exert phenotype-specific effects on survival and behavior. For example, variation in freshwater outflow is known to affect dispersal, habitat suitability, and population dynamics of several species (Jassby *et al.* 1995; Kimmerer 2002). Laboratory studies (Swanson *et al.* 2000; Feyrer *et al.* 2007; Nobriga *et al.* 2008; Komoroske *et al.* 2014, 2015; Jeffries *et al.* 2016) and observations of the wild population of Delta Smelt (Lewis *et al.* 2021; Hammock *et al.* 2022) indicate strong sensitivity to variation in temperature. In addition to longitudinal studies of life history traits, studies examining the degree to which genotypic and phenotypic variation corresponds with variation in the sensitivity of Delta Smelt to environmental variation remains an unexplored direction of inquiry (Lewis *et al.* 2022).

## Data availability

Sample metadata and scripts for analysis and generation of figures in this manuscript are available at https://github.com/MacCampbell/delta-smelt. Sequence data have been deposited with NCBI into the Sequence Read Archive under BioProject PRJNA834474. Otolith microchemistry profiles and assignments are available as part of the Bureau of Reclamation Directed Outflow Technical Report #3 (https://www.usbr.gov/mp/bdo/directed-outflow.html).

Supplemental material is available at *G3* online.

## Supplementary Material

jkac133_Supplementary_Document_S1

jkac133_Supplementary_Document_S2

jkac133_Supplementary_Document_S3

jkac133_Supplementary_Document_S4

jkac133_Supplementary_Figure_S1

jkac133_Supplementary_File_S1

jkac133_Supplementary_Appendix
