# Supplementary material for: Polygenic discrimination of migratory phenotypes in an estuarine forage fish
Source: G3 (Bethesda). 2022 May 30;12(8):jkac133. doi: 10.1093/g3journal/jkac133 (PMC9339312; doi:10.1093/g3journal/jkac133)
Supplement: jkac133_Supplementary_Document_S4 [file jkac133_supplementary_document_s4.zip › Supplemental_Document_S4_G3-2022-403487.html]

Supplemental Document S4


# Supplemental Document S4

#### Mac Campbell

#### 4/22/2022

```
library(tidyverse)
library(biomaRt)
library(knitr)
```

## Features Near Associated SNPs

Want chroms 1, 2, 15, and 23. Reading GFF from GenBank.

```
gff<-read_tsv("outputs/supplemental-document-s4/GCF_021917145.1_fHypTra1_genomic.gff.gz", skip=8, col_names = FALSE) %>% filter(X1 %in% c("NC_061060.1","NC_061061.1","NC_061074.1","NC_061082.1"))
```

```
genes<-filter(gff,X3=="gene")
```

What kind of linkage window do we have?  
https://speciationgenomics.github.io/ld\_decay/  
https://github.com/speciationgenomics/scripts/blob/master/ld\_decay\_calc.py

```
plink --vcf recode.vcf --allow-extra-chr --maf 0.05 --geno 0.1 --mind 0.5 --chr lg01 \
--thin 0.5 -r2 gz --ld-window 100000 --ld-window-kb 1000 \
--ld-window-r2 0 \
--make-bed --out dsm-lg01


source activate py2; ./ld_decay_calc.py -i dsm-lg01.ld.gz -o dsm-log01; conda deactivate;
```

```
ld_bins <- read_tsv("outputs/supplemental-document-s4/dsm-lg01.ld_decay_bins")

# plot LD decay
ggplot(ld_bins, aes(distance, avg_R2)) +
  geom_line() +
  xlab("Distance (bp)") + 
  ylab(expression(italic(r)^2)) +
  theme_bw() +
  theme(axis.text.x=element_text(angle=45, vjust=0.5))
```

```
ggplot(ld_bins, aes(distance, avg_R2)) +
  geom_line() +
  xlab("Distance (bp)") + 
  ylab(expression(italic(r)^2)) +
  theme_bw() +
  theme(axis.text.x=element_text(angle=45, vjust=0.5)) +
  xlim(0,200000)
```

```
ld_bins
```

```
## # A tibble: 49 × 4
##    chr   distance    avg_R2       std
##    <chr>    <dbl>     <dbl>     <dbl>
##  1 lg01       500 0.272     0.421    
##  2 lg01     26500 0.000859  0        
##  3 lg01     43500 0.00218   0        
##  4 lg01     56500 0         0        
##  5 lg01    150500 0.0172    0.0170   
##  6 lg01    159500 0.0115    0        
##  7 lg01    172500 0.00112   0        
##  8 lg01    182500 0.0250    0        
##  9 lg01    183500 0.0000168 0.0000128
## 10 lg01    208500 0.00812   0        
## # … with 39 more rows
```

By 26500 we’ve lost all ld.

```
genes %>% filter(X1=="NC_061060.1") %>% filter(X5 > 2467271-26500) %>% filter(X4 < 2467271+26500) %>% mutate( Distance = X4 - 2467271, End = X5 - 2467271)
```

```
## # A tibble: 3 × 11
##   X1          X2     X3         X4     X5 X6    X7    X8    X9    Distance   End
##   <chr>       <chr>  <chr>   <dbl>  <dbl> <chr> <chr> <chr> <chr>    <dbl> <dbl>
## 1 NC_061060.1 Gnomon gene  2437190 2.46e6 .     -     .     ID=g…   -30081 -5252
## 2 NC_061060.1 Gnomon gene  2474241 2.48e6 .     +     .     ID=g…     6970 12770
## 3 NC_061060.1 Gnomon gene  2480847 2.50e6 .     -     .     ID=g…    13576 30542
```

Transcfription factor

```
genes %>% filter(X1=="NC_061061.1") %>% filter(X5 > 11230311-26500) %>% filter(X4 < 11230464+26500)
```

```
## # A tibble: 3 × 9
##   X1          X2     X3          X4       X5 X6    X7    X8    X9               
##   <chr>       <chr>  <chr>    <dbl>    <dbl> <chr> <chr> <chr> <chr>            
## 1 NC_061061.1 Gnomon gene  11219956 11224590 .     +     .     ID=gene-nrsn1;Db…
## 2 NC_061061.1 Gnomon gene  11234513 11239105 .     -     .     ID=gene-LOC12447…
## 3 NC_061061.1 Gnomon gene  11243757 11253748 .     -     .     ID=gene-slc6a3;D…
```

nrsn1: nervous system development (GO:0007399) slc6a3: neurotransmitter transport

```
genes %>% filter(X1=="NC_061074.1") %>% filter(X5 > 2268817-26500) %>% filter(X4 < 2268817+26500)
```

```
## # A tibble: 12 × 9
##    X1          X2       X3         X4      X5 X6    X7    X8    X9              
##    <chr>       <chr>    <chr>   <dbl>   <dbl> <chr> <chr> <chr> <chr>           
##  1 NC_061074.1 Gnomon   gene  2238325 2243123 .     -     .     ID=gene-LOC1244…
##  2 NC_061074.1 Gnomon   gene  2255221 2257416 .     +     .     ID=gene-LOC1244…
##  3 NC_061074.1 Gnomon   gene  2261150 2266523 .     +     .     ID=gene-aamdc;D…
##  4 NC_061074.1 Gnomon   gene  2266390 2274233 .     -     .     ID=gene-ints4;D…
##  5 NC_061074.1 Gnomon   gene  2274295 2276264 .     -     .     ID=gene-kctd14;…
##  6 NC_061074.1 Gnomon   gene  2276521 2279995 .     -     .     ID=gene-rps3;Db…
##  7 NC_061074.1 cmsearch gene  2276708 2276854 .     -     .     ID=gene-LOC1244…
##  8 NC_061074.1 cmsearch gene  2277460 2277604 .     -     .     ID=gene-LOC1244…
##  9 NC_061074.1 cmsearch gene  2278135 2278281 .     -     .     ID=gene-LOC1244…
## 10 NC_061074.1 cmsearch gene  2278681 2278825 .     -     .     ID=gene-LOC1244…
## 11 NC_061074.1 Gnomon   gene  2280471 2286961 .     -     .     ID=gene-LOC1244…
## 12 NC_061074.1 Gnomon   gene  2287041 2317148 .     -     .     ID=gene-LOC1244…
```

```
genes %>% filter(X1=="NC_061082.1") %>% filter(X5 > 7996307-26500) %>% filter(X4 < 7996307+26500)
```

```
## # A tibble: 3 × 9
##   X1          X2     X3         X4      X5 X6    X7    X8    X9                 
##   <chr>       <chr>  <chr>   <dbl>   <dbl> <chr> <chr> <chr> <chr>              
## 1 NC_061082.1 Gnomon gene  7972427 7978876 .     -     .     ID=gene-LOC1244854…
## 2 NC_061082.1 Gnomon gene  7979005 7986497 .     -     .     ID=gene-tfcp2l1;Db…
## 3 NC_061082.1 Gnomon gene  7987047 8045995 .     -     .     ID=gene-clasp1a;Db…
```

Get GO’s

```
ensembl = useEnsembl(biomart="genes", mirror="uswest", dataset = "drerio_gene_ensembl")
```

```
geneDat <- getBM(attributes=c('description', 'zfin_id_symbol','ensembl_gene_id','go_id','name_1006',"definition_1006"),
              mart = ensembl, filters='zfin_id_symbol', 
              values=c("nrsn1","nfkb1","slc6a3","aamdc","ints4","kctd14","rps3","tfcp2l1","clasp1a"))
```

```
kable(geneDat, caption="biomaRt results")
```

biomaRt results


| description | zfin\_id\_symbol | ensembl\_gene\_id | go\_id | name\_1006 | definition\_1006 |
| --- | --- | --- | --- | --- | --- |
| adipogenesis associated, Mth938 domain containing [Source:NCBI gene;Acc:553802] | aamdc | ENSDARG00000103957 |  |  |  |
| cytoplasmic linker associated protein 1a [Source:ZFIN;Acc:ZDB-GENE-081104-520] | clasp1a | ENSDARG00000010280 | GO:0008017 | microtubule binding | Binding to a microtubule, a filament composed of tubulin monomers. |
| cytoplasmic linker associated protein 1a [Source:ZFIN;Acc:ZDB-GENE-081104-520] | clasp1a | ENSDARG00000010280 | GO:0043515 | kinetochore binding | Binding to a kinetochore, a proteinaceous structure on a condensed chromosome, beside the centromere, to which the spindle fibers are attached. |
| cytoplasmic linker associated protein 1a [Source:ZFIN;Acc:ZDB-GENE-081104-520] | clasp1a | ENSDARG00000010280 | GO:0051010 | microtubule plus-end binding | Binding to the plus end of a microtubule. |
| cytoplasmic linker associated protein 1a [Source:ZFIN;Acc:ZDB-GENE-081104-520] | clasp1a | ENSDARG00000010280 | GO:0005828 | kinetochore microtubule | Any of the spindle microtubules that attach to the kinetochores of chromosomes by their plus ends, and maneuver the chromosomes during mitotic or meiotic chromosome segregation. |
| cytoplasmic linker associated protein 1a [Source:ZFIN;Acc:ZDB-GENE-081104-520] | clasp1a | ENSDARG00000010280 | GO:0005881 | cytoplasmic microtubule | Any microtubule in the cytoplasm of a cell. |
| cytoplasmic linker associated protein 1a [Source:ZFIN;Acc:ZDB-GENE-081104-520] | clasp1a | ENSDARG00000010280 | GO:0000226 | microtubule cytoskeleton organization | A process that is carried out at the cellular level which results in the assembly, arrangement of constituent parts, or disassembly of cytoskeletal structures comprising microtubules and their associated proteins. |
| cytoplasmic linker associated protein 1a [Source:ZFIN;Acc:ZDB-GENE-081104-520] | clasp1a | ENSDARG00000010280 | GO:0005815 | microtubule organizing center | An intracellular structure that can catalyze gamma-tubulin-dependent microtubule nucleation and that can anchor microtubules by interacting with their minus ends, plus ends or sides. |
| cytoplasmic linker associated protein 1a [Source:ZFIN;Acc:ZDB-GENE-081104-520] | clasp1a | ENSDARG00000010280 | GO:0000776 | kinetochore | A multisubunit complex that is located at the centromeric region of DNA and provides an attachment point for the spindle microtubules. |
| cytoplasmic linker associated protein 1a [Source:ZFIN;Acc:ZDB-GENE-081104-520] | clasp1a | ENSDARG00000010280 | GO:0072686 | mitotic spindle | A spindle that forms as part of mitosis. Mitotic and meiotic spindles contain distinctive complements of proteins associated with microtubules. |
| cytoplasmic linker associated protein 1a [Source:ZFIN;Acc:ZDB-GENE-081104-520] | clasp1a | ENSDARG00000010280 | GO:0090307 | mitotic spindle assembly | Mitotic bipolar spindle assembly begins with spindle microtubule nucleation from the separated spindle pole body, includes spindle elongation during prometaphase, and is complete when all kinetochores are stably attached the spindle, and the spindle assembly checkpoint is satisfied. |
| cytoplasmic linker associated protein 1a [Source:ZFIN;Acc:ZDB-GENE-081104-520] | clasp1a | ENSDARG00000010280 | GO:0045180 | basal cortex | The region that lies just beneath the plasma membrane on the basal edge of a cell. |
| cytoplasmic linker associated protein 1a [Source:ZFIN;Acc:ZDB-GENE-081104-520] | clasp1a | ENSDARG00000010280 | GO:0005876 | spindle microtubule | Any microtubule that is part of a mitotic or meiotic spindle; anchored at one spindle pole. |
| cytoplasmic linker associated protein 1a [Source:ZFIN;Acc:ZDB-GENE-081104-520] | clasp1a | ENSDARG00000010280 | GO:0040001 | establishment of mitotic spindle localization | The cell cycle process in which the directed movement of the mitotic spindle to a specific location in the cell occurs. |
| cytoplasmic linker associated protein 1a [Source:ZFIN;Acc:ZDB-GENE-081104-520] | clasp1a | ENSDARG00000010280 | GO:0005794 | Golgi apparatus | A membrane-bound cytoplasmic organelle of the endomembrane system that further processes the core oligosaccharides (e.g. N-glycans) added to proteins in the endoplasmic reticulum and packages them into membrane-bound vesicles. The Golgi apparatus operates at the intersection of the secretory, lysosomal, and endocytic pathways. |
| integrator complex subunit 4 [Source:ZFIN;Acc:ZDB-GENE-120215-81] | ints4 | ENSDARG00000102953 |  |  |  |
| potassium channel tetramerization domain containing 14 [Source:ZFIN;Acc:ZDB-GENE-120215-239] | kctd14 | ENSDARG00000110240 | GO:0051260 | protein homooligomerization | The process of creating protein oligomers, compounds composed of a small number, usually between three and ten, of identical component monomers. Oligomers may be formed by the polymerization of a number of monomers or the depolymerization of a large protein polymer. |
| potassium channel tetramerization domain containing 14 [Source:ZFIN;Acc:ZDB-GENE-120215-239] | kctd14 | ENSDARG00000110240 | GO:0005515 | protein binding | Binding to a protein. |
| nuclear factor of kappa light polypeptide gene enhancer in B-cells 1 [Source:ZFIN;Acc:ZDB-GENE-121204-3] | nfkb1 | ENSDARG00000105261 | GO:0006357 | regulation of transcription by RNA polymerase II | Any process that modulates the frequency, rate or extent of transcription mediated by RNA polymerase II. |
| nuclear factor of kappa light polypeptide gene enhancer in B-cells 1 [Source:ZFIN;Acc:ZDB-GENE-121204-3] | nfkb1 | ENSDARG00000105261 | GO:0003700 | DNA-binding transcription factor activity | A transcription regulator activity that modulates transcription of gene sets via selective and non-covalent binding to a specific double-stranded genomic DNA sequence (sometimes referred to as a motif) within a cis-regulatory region. Regulatory regions include promoters (proximal and distal) and enhancers. Genes are transcriptional units, and include bacterial operons. |
| nuclear factor of kappa light polypeptide gene enhancer in B-cells 1 [Source:ZFIN;Acc:ZDB-GENE-121204-3] | nfkb1 | ENSDARG00000105261 | GO:0005737 | cytoplasm | The contents of a cell excluding the plasma membrane and nucleus, but including other subcellular structures. |
| nuclear factor of kappa light polypeptide gene enhancer in B-cells 1 [Source:ZFIN;Acc:ZDB-GENE-121204-3] | nfkb1 | ENSDARG00000105261 | GO:0005634 | nucleus | A membrane-bounded organelle of eukaryotic cells in which chromosomes are housed and replicated. In most cells, the nucleus contains all of the cell’s chromosomes except the organellar chromosomes, and is the site of RNA synthesis and processing. In some species, or in specialized cell types, RNA metabolism or DNA replication may be absent. |
| nuclear factor of kappa light polypeptide gene enhancer in B-cells 1 [Source:ZFIN;Acc:ZDB-GENE-121204-3] | nfkb1 | ENSDARG00000105261 | GO:0006355 | regulation of transcription, DNA-templated | Any process that modulates the frequency, rate or extent of cellular DNA-templated transcription. |
| nuclear factor of kappa light polypeptide gene enhancer in B-cells 1 [Source:ZFIN;Acc:ZDB-GENE-121204-3] | nfkb1 | ENSDARG00000105261 | GO:0003677 | DNA binding | Any molecular function by which a gene product interacts selectively and non-covalently with DNA (deoxyribonucleic acid). |
| neurensin 1 [Source:ZFIN;Acc:ZDB-GENE-030131-7491] | nrsn1 | ENSDARG00000111235 | GO:0016020 | membrane | A lipid bilayer along with all the proteins and protein complexes embedded in it an attached to it. |
| neurensin 1 [Source:ZFIN;Acc:ZDB-GENE-030131-7491] | nrsn1 | ENSDARG00000111235 | GO:0016021 | integral component of membrane | The component of a membrane consisting of the gene products and protein complexes having at least some part of their peptide sequence embedded in the hydrophobic region of the membrane. |
| neurensin 1 [Source:ZFIN;Acc:ZDB-GENE-030131-7491] | nrsn1 | ENSDARG00000111235 | GO:0043005 | neuron projection | A prolongation or process extending from a nerve cell, e.g. an axon or dendrite. |
| neurensin 1 [Source:ZFIN;Acc:ZDB-GENE-030131-7491] | nrsn1 | ENSDARG00000111235 | GO:0003674 | molecular\_function | A molecular process that can be carried out by the action of a single macromolecular machine, usually via direct physical interactions with other molecular entities. Function in this sense denotes an action, or activity, that a gene product (or a complex) performs. These actions are described from two distinct but related perspectives: (1) biochemical activity, and (2) role as a component in a larger system/process. |
| neurensin 1 [Source:ZFIN;Acc:ZDB-GENE-030131-7491] | nrsn1 | ENSDARG00000111235 | GO:0007399 | nervous system development | The process whose specific outcome is the progression of nervous tissue over time, from its formation to its mature state. |
| neurensin 1 [Source:ZFIN;Acc:ZDB-GENE-030131-7491] | nrsn1 | ENSDARG00000111235 | GO:0043025 | neuronal cell body | The portion of a neuron that includes the nucleus, but excludes cell projections such as axons and dendrites. |
| neurensin 1 [Source:ZFIN;Acc:ZDB-GENE-030131-7491] | nrsn1 | ENSDARG00000111235 | GO:0030133 | transport vesicle | Any of the vesicles of the constitutive secretory pathway, which carry cargo from the endoplasmic reticulum to the Golgi, between Golgi cisternae, from the Golgi to the ER (retrograde transport) or to destinations within or outside the cell. |
| ribosomal protein S3 [Source:ZFIN;Acc:ZDB-GENE-030131-8494] | rps3 | ENSDARG00000103007 | GO:0016020 | membrane | A lipid bilayer along with all the proteins and protein complexes embedded in it an attached to it. |
| ribosomal protein S3 [Source:ZFIN;Acc:ZDB-GENE-030131-8494] | rps3 | ENSDARG00000103007 | GO:0005737 | cytoplasm | The contents of a cell excluding the plasma membrane and nucleus, but including other subcellular structures. |
| ribosomal protein S3 [Source:ZFIN;Acc:ZDB-GENE-030131-8494] | rps3 | ENSDARG00000103007 | GO:0043009 | chordate embryonic development | The process whose specific outcome is the progression of the embryo over time, from zygote formation through a stage including a notochord and neural tube until birth or egg hatching. |
| ribosomal protein S3 [Source:ZFIN;Acc:ZDB-GENE-030131-8494] | rps3 | ENSDARG00000103007 | GO:0005634 | nucleus | A membrane-bounded organelle of eukaryotic cells in which chromosomes are housed and replicated. In most cells, the nucleus contains all of the cell’s chromosomes except the organellar chromosomes, and is the site of RNA synthesis and processing. In some species, or in specialized cell types, RNA metabolism or DNA replication may be absent. |
| ribosomal protein S3 [Source:ZFIN;Acc:ZDB-GENE-030131-8494] | rps3 | ENSDARG00000103007 | GO:0003723 | RNA binding | Binding to an RNA molecule or a portion thereof. |
| ribosomal protein S3 [Source:ZFIN;Acc:ZDB-GENE-030131-8494] | rps3 | ENSDARG00000103007 | GO:0005743 | mitochondrial inner membrane | The inner, i.e. lumen-facing, lipid bilayer of the mitochondrial envelope. It is highly folded to form cristae. |
| ribosomal protein S3 [Source:ZFIN;Acc:ZDB-GENE-030131-8494] | rps3 | ENSDARG00000103007 | GO:0006915 | apoptotic process | A programmed cell death process which begins when a cell receives an internal (e.g. DNA damage) or external signal (e.g. an extracellular death ligand), and proceeds through a series of biochemical events (signaling pathway phase) which trigger an execution phase. The execution phase is the last step of an apoptotic process, and is typically characterized by rounding-up of the cell, retraction of pseudopodes, reduction of cellular volume (pyknosis), chromatin condensation, nuclear fragmentation (karyorrhexis), plasma membrane blebbing and fragmentation of the cell into apoptotic bodies. When the execution phase is completed, the cell has died. |
| ribosomal protein S3 [Source:ZFIN;Acc:ZDB-GENE-030131-8494] | rps3 | ENSDARG00000103007 | GO:0016829 | lyase activity | Catalysis of the cleavage of C-C, C-O, C-N and other bonds by other means than by hydrolysis or oxidation, or conversely adding a group to a double bond. They differ from other enzymes in that two substrates are involved in one reaction direction, but only one in the other direction. When acting on the single substrate, a molecule is eliminated and this generates either a new double bond or a new ring. |
| ribosomal protein S3 [Source:ZFIN;Acc:ZDB-GENE-030131-8494] | rps3 | ENSDARG00000103007 | GO:0003735 | structural constituent of ribosome | The action of a molecule that contributes to the structural integrity of the ribosome. |
| ribosomal protein S3 [Source:ZFIN;Acc:ZDB-GENE-030131-8494] | rps3 | ENSDARG00000103007 | GO:0006412 | translation | The cellular metabolic process in which a protein is formed, using the sequence of a mature mRNA or circRNA molecule to specify the sequence of amino acids in a polypeptide chain. Translation is mediated by the ribosome, and begins with the formation of a ternary complex between aminoacylated initiator methionine tRNA, GTP, and initiation factor 2, which subsequently associates with the small subunit of the ribosome and an mRNA or circRNA. Translation ends with the release of a polypeptide chain from the ribosome. |
| ribosomal protein S3 [Source:ZFIN;Acc:ZDB-GENE-030131-8494] | rps3 | ENSDARG00000103007 | GO:0030218 | erythrocyte differentiation | The process in which a myeloid precursor cell acquires specializes features of an erythrocyte. |
| ribosomal protein S3 [Source:ZFIN;Acc:ZDB-GENE-030131-8494] | rps3 | ENSDARG00000103007 | GO:0051301 | cell division | The process resulting in division and partitioning of components of a cell to form more cells; may or may not be accompanied by the physical separation of a cell into distinct, individually membrane-bounded daughter cells. |
| ribosomal protein S3 [Source:ZFIN;Acc:ZDB-GENE-030131-8494] | rps3 | ENSDARG00000103007 | GO:0005840 | ribosome | An intracellular organelle, about 200 A in diameter, consisting of RNA and protein. It is the site of protein biosynthesis resulting from translation of messenger RNA (mRNA). It consists of two subunits, one large and one small, each containing only protein and RNA. Both the ribosome and its subunits are characterized by their sedimentation coefficients, expressed in Svedberg units (symbol: S). Hence, the prokaryotic ribosome (70S) comprises a large (50S) subunit and a small (30S) subunit, while the eukaryotic ribosome (80S) comprises a large (60S) subunit and a small (40S) subunit. Two sites on the ribosomal large subunit are involved in translation, namely the aminoacyl site (A site) and peptidyl site (P site). Ribosomes from prokaryotes, eukaryotes, mitochondria, and chloroplasts have characteristically distinct ribosomal proteins. |
| ribosomal protein S3 [Source:ZFIN;Acc:ZDB-GENE-030131-8494] | rps3 | ENSDARG00000103007 | GO:0006281 | DNA repair | The process of restoring DNA after damage. Genomes are subject to damage by chemical and physical agents in the environment (e.g. UV and ionizing radiations, chemical mutagens, fungal and bacterial toxins, etc.) and by free radicals or alkylating agents endogenously generated in metabolism. DNA is also damaged because of errors during its replication. A variety of different DNA repair pathways have been reported that include direct reversal, base excision repair, nucleotide excision repair, photoreactivation, bypass, double-strand break repair pathway, and mismatch repair pathway. |
| ribosomal protein S3 [Source:ZFIN;Acc:ZDB-GENE-030131-8494] | rps3 | ENSDARG00000103007 | GO:0006974 | cellular response to DNA damage stimulus | Any process that results in a change in state or activity of a cell (in terms of movement, secretion, enzyme production, gene expression, etc.) as a result of a stimulus indicating damage to its DNA from environmental insults or errors during metabolism. |
| ribosomal protein S3 [Source:ZFIN;Acc:ZDB-GENE-030131-8494] | rps3 | ENSDARG00000103007 | GO:0005819 | spindle | The array of microtubules and associated molecules that forms between opposite poles of a eukaryotic cell during mitosis or meiosis and serves to move the duplicated chromosomes apart. |
| ribosomal protein S3 [Source:ZFIN;Acc:ZDB-GENE-030131-8494] | rps3 | ENSDARG00000103007 | GO:0022627 | cytosolic small ribosomal subunit | The small subunit of a ribosome located in the cytosol. |
| ribosomal protein S3 [Source:ZFIN;Acc:ZDB-GENE-030131-8494] | rps3 | ENSDARG00000103007 | GO:0140078 | class I DNA-(apurinic or apyrimidinic site) endonuclease activity | Catalysis of the cleavage of an AP site 3’ of the baseless site by a beta-lyase mechanism, leaving an unsaturated aldehyde, termed a 3’-(4-hydroxy-5-phospho-2-pentenal) residue, and a 5’-phosphate. |
| ribosomal protein S3 [Source:ZFIN;Acc:ZDB-GENE-030131-8494] | rps3 | ENSDARG00000103007 | GO:0015935 | small ribosomal subunit | The smaller of the two subunits of a ribosome. |
| ribosomal protein S3 [Source:ZFIN;Acc:ZDB-GENE-030131-8494] | rps3 | ENSDARG00000103007 | GO:0006417 | regulation of translation | Any process that modulates the frequency, rate or extent of the chemical reactions and pathways resulting in the formation of proteins by the translation of mRNA or circRNA. |
| ribosomal protein S3 [Source:ZFIN;Acc:ZDB-GENE-030131-8494] | rps3 | ENSDARG00000103007 | GO:2001235 | positive regulation of apoptotic signaling pathway | Any process that activates or increases the frequency, rate or extent of apoptotic signaling pathway. |
| solute carrier family 6 member 3 [Source:ZFIN;Acc:ZDB-GENE-010316-1] | slc6a3 | ENSDARG00000004219 | GO:0016020 | membrane | A lipid bilayer along with all the proteins and protein complexes embedded in it an attached to it. |
| solute carrier family 6 member 3 [Source:ZFIN;Acc:ZDB-GENE-010316-1] | slc6a3 | ENSDARG00000004219 | GO:0016021 | integral component of membrane | The component of a membrane consisting of the gene products and protein complexes having at least some part of their peptide sequence embedded in the hydrophobic region of the membrane. |
| solute carrier family 6 member 3 [Source:ZFIN;Acc:ZDB-GENE-010316-1] | slc6a3 | ENSDARG00000004219 | GO:0005887 | integral component of plasma membrane | The component of the plasma membrane consisting of the gene products and protein complexes having at least some part of their peptide sequence embedded in the hydrophobic region of the membrane. |
| solute carrier family 6 member 3 [Source:ZFIN;Acc:ZDB-GENE-010316-1] | slc6a3 | ENSDARG00000004219 | GO:0043005 | neuron projection | A prolongation or process extending from a nerve cell, e.g. an axon or dendrite. |
| solute carrier family 6 member 3 [Source:ZFIN;Acc:ZDB-GENE-010316-1] | slc6a3 | ENSDARG00000004219 | GO:0015293 | symporter activity | Enables the active transport of a solute across a membrane by a mechanism whereby two or more species are transported together in the same direction in a tightly coupled process not directly linked to a form of energy other than chemiosmotic energy. |
| solute carrier family 6 member 3 [Source:ZFIN;Acc:ZDB-GENE-010316-1] | slc6a3 | ENSDARG00000004219 | GO:0035725 | sodium ion transmembrane transport | A process in which a sodium ion is transported from one side of a membrane to the other by means of some agent such as a transporter or pore. |
| solute carrier family 6 member 3 [Source:ZFIN;Acc:ZDB-GENE-010316-1] | slc6a3 | ENSDARG00000004219 | GO:0042734 | presynaptic membrane | A specialized area of membrane of the axon terminal that faces the plasma membrane of the neuron or muscle fiber with which the axon terminal establishes a synaptic junction; many synaptic junctions exhibit structural presynaptic characteristics, such as conical, electron-dense internal protrusions, that distinguish it from the remainder of the axon plasma membrane. |
| solute carrier family 6 member 3 [Source:ZFIN;Acc:ZDB-GENE-010316-1] | slc6a3 | ENSDARG00000004219 | GO:0032809 | neuronal cell body membrane | The plasma membrane of a neuron cell body - excludes the plasma membrane of cell projections such as axons and dendrites. |
| solute carrier family 6 member 3 [Source:ZFIN;Acc:ZDB-GENE-010316-1] | slc6a3 | ENSDARG00000004219 | GO:0005330 | dopamine:sodium symporter activity | Enables the transfer of a solute or solutes from one side of a membrane to the other according to the reaction: dopamine(out) + Na+(out) + Cl-(out)= dopamine(in) + Na+(in) + Cl-(in). |
| solute carrier family 6 member 3 [Source:ZFIN;Acc:ZDB-GENE-010316-1] | slc6a3 | ENSDARG00000004219 | GO:0005334 | norepinephrine:sodium symporter activity | Enables the transfer of a solute or solutes from one side of a membrane to the other according to the reaction: norepinephrine(out) + Na+(out) + Cl-(out) = norepinephrine(in) + Na+(in) + Cl-(in). |
| solute carrier family 6 member 3 [Source:ZFIN;Acc:ZDB-GENE-010316-1] | slc6a3 | ENSDARG00000004219 | GO:0015874 | norepinephrine transport | The directed movement of norepinephrine into, out of or within a cell, or between cells, by means of some agent such as a transporter or pore. Norepinephrine (3,4-dihydroxyphenyl-2-aminoethanol) is a hormone secreted by the adrenal medulla and a neurotransmitter in the sympathetic peripheral nervous system and in some tracts of the CNS. It is also the biosynthetic precursor of epinephrine. |
| solute carrier family 6 member 3 [Source:ZFIN;Acc:ZDB-GENE-010316-1] | slc6a3 | ENSDARG00000004219 | GO:0051583 | dopamine uptake involved in synaptic transmission | The directed movement of dopamine into a presynaptic neuron or glial cell. In this context, dopamine is a catecholamine neurotransmitter and a metabolic precursor of noradrenaline and adrenaline. |
| solute carrier family 6 member 3 [Source:ZFIN;Acc:ZDB-GENE-010316-1] | slc6a3 | ENSDARG00000004219 | GO:0051620 | norepinephrine uptake | The directed movement of norepinephrine into a cell, typically presynaptic neurons or glial cells. Norepinephrine (3,4-dihydroxyphenyl-2-aminoethanol) is a hormone secreted by the adrenal medulla and a neurotransmitter in the sympathetic peripheral nervous system and in some tracts of the CNS. It is also the biosynthetic precursor of epinephrine. |
| transcription factor CP2-like 1 [Source:ZFIN;Acc:ZDB-GENE-040704-58] | tfcp2l1 | ENSDARG00000029497 |  |  |  |
| transcription factor CP2-like 1 [Source:ZFIN;Acc:ZDB-GENE-040704-58] | tfcp2l1 | ENSDARG00000029497 | GO:0006357 | regulation of transcription by RNA polymerase II | Any process that modulates the frequency, rate or extent of transcription mediated by RNA polymerase II. |
| transcription factor CP2-like 1 [Source:ZFIN;Acc:ZDB-GENE-040704-58] | tfcp2l1 | ENSDARG00000029497 | GO:0003700 | DNA-binding transcription factor activity | A transcription regulator activity that modulates transcription of gene sets via selective and non-covalent binding to a specific double-stranded genomic DNA sequence (sometimes referred to as a motif) within a cis-regulatory region. Regulatory regions include promoters (proximal and distal) and enhancers. Genes are transcriptional units, and include bacterial operons. |

```
write_csv(geneDat, file="outputs/supplemental-document-s4/go-terms.csv")
```

```
geneDat %>% as_tibble() %>% dplyr::select(go_id) %>% write_tsv("outputs/supplemental-document-s4/gos.tsv")
```
