# Supplementary material for: Polygenic discrimination of migratory phenotypes in an estuarine forage fish
Source: G3 (Bethesda). 2022 May 30;12(8):jkac133. doi: 10.1093/g3journal/jkac133 (PMC9339312; doi:10.1093/g3journal/jkac133)
Supplement: jkac133_Supplementary_Figure_S1 [file jkac133_supplementary_figure_s1.docx]

**Supplemental Figure S1** Heatmap showing clustering of Delta Smelt based on the 200 most-associated genotypes with life-history variation. Individual fishes are represented by rows and color-coded by freshwater resident (FWR) as red and semi-anadrmous (MIG) as blue. Rows are genotypes, color coded as yellow for homozygous minor, green for heterozygous, and purple for homozygous major. Both rows and columns were clustered with Ward’s distance, with the dendrogram for columns not shown.
