# Supplementary material for: Polygenic discrimination of migratory phenotypes in an estuarine forage fish
Source: G3 (Bethesda). 2022 May 30;12(8):jkac133. doi: 10.1093/g3journal/jkac133 (PMC9339312; doi:10.1093/g3journal/jkac133)
Supplement: jkac133_Supplementary_Appendix [file jkac133_supplementary_appendix.zip › Appendix_1_G3-2022-403487.html]

Appendix-1


# Appendix-1

#### Mac Campbell

#### 4/21/2022

```
library(tidyverse)
library(vcfR)
library(adegenet)
library(ggpubr)
library(snpR)
library(BSDA)
library(ggmap)
```

## Prepare Meta

```
meta<-read_csv("metadata/2012-samples.csv")

write_tsv(meta %>% select(Sample_ID), col_names = FALSE, file="outputs/appendix-1/names.tsv")
```

## I RAD loci

**1** Calculate RAD loci based on regex for ballpark, then in silico prediction

```
grep -o  -i CCTGCAGG large-contigs.fasta | wc -l
```

14,673

Reverse complement

```
grep -o  -i GGACGTCC large-contigs.fasta | wc -l
```

3,849

14673+3879 [1] 18552

In silico prediction  
`perl -I ~/perl5/lib/perl5/ ~/Dropbox/bin/restrictionAnalysis.pl SbfI large-contigs.fasta`

The total number of restriction sites for SbfI is: 16578  
The average fragment size is: 24047.7176145577  
The total number of fragments is: 16651  
The total number of genome contigs: 73

**2** RAD Loci based on GLs and called SNPs

In terms of GL’s, we can aggregrate

```
dist<-read_tsv("outputs/100/genolike.beagle.gz") %>% separate(marker,into=c("Chrom","Pos"))
temp<-dist %>% filter(startsWith(Chrom, "lg")) # %>% filter(Chrom %in% c("lg01","lg02"))
temp$Pos<-as.numeric(temp$Pos)
```

```
temp %>% group_by(Chrom) %>% mutate(trunc1 = (Pos %/% 5000) * 5000) %>% select(Chrom, Pos, trunc1) %>% select(Chrom, trunc1) %>% unique()
```

```
## # A tibble: 11,638 × 2
## # Groups:   Chrom [26]
##    Chrom trunc1
##    <chr>  <dbl>
##  1 lg01   25000
##  2 lg01   40000
##  3 lg01   45000
##  4 lg01   95000
##  5 lg01  100000
##  6 lg01  150000
##  7 lg01  170000
##  8 lg01  195000
##  9 lg01  200000
## 10 lg01  215000
## # … with 11,628 more rows
```

11,638 loci

In terms of calls, we can define loci:

https://darencard.net/blog/2019-12-20-RAD-locus-footprints/

```
cat ../../metadata/2012-samples.csv | cut -d ',' -f 4,29,38 | perl -pe 's/,/\t/g' > spreadsheet.txt             
cat ../../bamlists/2012.bamlist | perl -pe 's/bams\///g' | perl -pe 's/\.sort.+$//g' > list.txt          
cat list.txt | while read line; do grep $line spreadsheet.txt >> 2012.txt; done;      
cut -f 3 2012.txt | while read line; do ln -s ../../bams/$line.sort.flt.bam . ; done;      
cut -f 3 2012.txt | while read line; do ln -s ../../bams/$line.sort.flt.bam.bai . ; done;      
cat 2012.txt | awk ' {print $1, $2, $3, "combined"} ' > combined.txt        
cat combined.txt | perl -pe 's/ /\t/g' > combined-tab.txt      
srun -p high -t 4:00:00 bash script.sh combined-tab.txt 4 3 .sort.flt.bam 20 5 25 25 200      
srun -p high -t 4:00:00 bash script.sh combined-tab.txt 4 3 .sort.flt.bam 20 5 25 25 100
```

`maccamp@farm:~/delta-smelt/outputs/appendix-1$ wc -l combined_rad_loci.qual20.dp5.miss25.merge25.len100.bed`

11090 combined\_rad\_loci.qual20.dp5.miss25.merge25.len100.bed

`maccamp@farm:~/delta-smelt/outputs/appendix-1$ wc -l combined_rad_loci.qual20.dp5.miss25.merge25.len200.bed`

2674 combined\_rad\_loci.qual20.dp5.miss25.merge25.len200.bed

## II PCA of Pruned SNPs

pruning and reheadering vcf

```
bcftools +prune -l 0.6 -w 10000 recode.vcf  -Ov -o recode.prune.vcf
bcftools reheader --samples names.tv -o recode.prune.reheadered.vcf recode.prune.vcf
```

```
vcf<-read.vcfR(file="outputs/appendix-1/recode.prune.reheadered.vcf")
```

```
## Scanning file to determine attributes.
## File attributes:
##   meta lines: 75
##   header_line: 76
##   variant count: 7274
##   column count: 130
## 
Meta line 75 read in.
## All meta lines processed.
## gt matrix initialized.
## Character matrix gt created.
##   Character matrix gt rows: 7274
##   Character matrix gt cols: 130
##   skip: 0
##   nrows: 7274
##   row_num: 0
## 
Processed variant 1000
Processed variant 2000
Processed variant 3000
Processed variant 4000
Processed variant 5000
Processed variant 6000
Processed variant 7000
Processed variant: 7274
## All variants processed
```

```
genind<-vcfR2genind(vcf)
save(genind, file="outputs/appendix-1/reheadered.genind")
```

```
genind@pop=as.factor(meta$aggregated_new_classes)

gen<-genind
X <- tab(gen, NA.method="mean")

pca1 <- dudi.pca(X,scannf=FALSE,scale=FALSE, nf = 3)
temp <- as.integer(pop(gen))
myCol <- transp(c("red","blue"),.7)[temp]
myPch <- c(15,17)[temp]
plot(pca1$li, col=myCol, cex=1, pch=myPch)
```

Tidyversion

```
pcdf<-as_tibble(pca1$l1) %>% rename(PC1=RS1, PC2=RS2, PC3=RS3)
pcdf$Phenotype<-pop(gen)

eigens<-as_tibble(pca1$eig) %>% rename(Eigen=value) %>% mutate(Porportion=Eigen/sum(Eigen)*100)

pc12<-ggplot(pcdf)+
  geom_point(aes(x=PC1, y=PC2, fill=Phenotype), alpha=0.75, size=2, pch=21)+
  scale_fill_manual(values = c("red","blue")) +
  xlab(paste0("PC1 ", round(eigens$Porportion[1],2),"%")) +
  ylab(paste0("PC2 ", round(eigens$Porportion[2],2),"%")) +
  theme_bw()+
  theme(panel.grid=element_blank()) +
  theme(axis.title = element_text(face="bold")) +
  theme(legend.title = element_text(face="bold")) +
  theme(legend.position = "NONE")

pc13<-ggplot(pcdf)+
  geom_point(aes(x=PC1, y=PC3, fill=Phenotype), alpha=0.75, size=2, pch=21)+
  scale_fill_manual(values = c("red","blue")) +
  xlab(paste0("PC1 ", round(eigens$Porportion[1],2),"%")) +
  ylab(paste0("PC3 ", round(eigens$Porportion[3],2),"%")) +
  theme_bw()+
  theme(panel.grid=element_blank()) +
  theme(axis.title = element_text(face="bold")) +
  theme(legend.title = element_text(face="bold")) +
  theme(legend.position = "NONE")
```

```
pcs<-ggarrange(pc12, pc13, ncol = 2, widths=c(1,1.25))
pcs
```

```
ggsave("outputs/appendix-1/snpspcs.jpeg", width=8, height=3.5)
```

```
ggplot(pcdf)+
  geom_point(aes(x=PC1, y=PC2, fill=Phenotype), alpha=0.75, size=2, pch=21)+
  scale_fill_manual(values = c("red","blue")) +
  xlab(paste0("PC1 ", round(eigens$Porportion[1],2),"%")) +
  ylab(paste0("PC2 ", round(eigens$Porportion[2],2),"%")) +
  theme_bw()+
  theme(panel.grid=element_blank()) +
  theme(axis.title = element_text(face="bold")) +
  theme(legend.title = element_text(face="bold"))
```

```
ggsave("outputs/appendix-1/snpspcs12.jpeg", width=4.5, height=3.5)
```

## III Nucleotide Diversity

```
cols<-meta %>% select(aggregated_new_classes) %>% mutate(Color=ifelse(aggregated_new_classes=="MIG","blue","red"))

genos<-read_tsv("outputs/100/snps-characters.geno.gz", col_names=FALSE) %>% mutate(Site=paste0(X1,"-",X2))

pruned<-read_tsv("outputs/appendix-1/pruned-markers.txt", col_names = c("Chrom","Pos")) %>% mutate(Site=paste0(Chrom,"-",Pos))

genos<-filter(genos, Site %in% pruned$Site) %>% select(-Site)

genos<-genos[1:ncol(genos)-1]

genos$X1<-as.factor(genos$X1)
```

```
# grab our sample metadata
snp_meta <- genos[,1:2]
sample_meta<-select(meta, aggregated_new_classes) %>% rename(Phenotype=aggregated_new_classes)
sample_meta$Phenotype<-as.factor(sample_meta$Phenotype)
# import, remember to remove metadata from the genotypes!
my.dat <- import.snpR.data(genos[,-c(1:2)], 
                           snp.meta = snp_meta, 
                           sample.meta = sample_meta)
```

```
## Assuming data is in NN format.
## Getting allele table...
## Input data will be filtered to remove non bi-allelic data.
```

Plotting PCA for sanity:

```
p <- plot_clusters(my.dat, facets = c("Phenotype"))
```

```
## Formatting data...
## Converting to single character numeric format.
## Plotting using 7274 loci and 121 samples.
## Preparing pca...
```

```
x <- calc_basic_snp_stats(my.dat, "Phenotype")
```

```
## Collating results...
```

```
stats<-get.snpR.stats(x, "Phenotype", stats="single")
```

```
ggplot(stats) +
  geom_boxplot(aes(y=pi, fill=subfacet)) +
  facet_wrap(.~subfacet) +
  theme_bw()+
  theme(panel.grid=element_blank()) +
  scale_fill_manual("Phenotype", values=c(rgb(1,0,0,0.75),rgb(0,0,1,0.75)))
```

```
v1<-stats %>% filter(subfacet=="FWR") %>% select(pi)
v2<-stats %>% filter(subfacet=="MIG") %>% select(pi)
```

```
t.test(v1, v2)
```

```
## 
##  Welch Two Sample t-test
## 
## data:  v1 and v2
## t = 0.89969, df = 14546, p-value = 0.3683
## alternative hypothesis: true difference in means is not equal to 0
## 95 percent confidence interval:
##  -0.002441991  0.006585602
## sample estimates:
## mean of x mean of y 
## 0.2211018 0.2190300
```

## IV Length and Sex

Length arranged by sample date

```
mm<-meta %>% select(Sample_ID, Year, SampleDate, `Fork Length`, new_classes, station, lat, lon) %>% filter(Year==2013) %>%
  filter(new_classes %in% c("FWR1", "FWM1", "FWM2")) %>%
  mutate(Phenotype=ifelse(new_classes=="FWR1","FWR","MIG")) %>%
  separate(SampleDate, c("Day","Month","Year"))
mm$Day<-as.numeric(mm$Day)
mm$Year<-as.numeric(mm$Year)
mm$Month<-factor(mm$Month, levels = c("Jan","Feb","Mar"))
```

```
mm %>% group_by(Phenotype, Month) %>% summarize(Count=n()) %>% select(Phenotype, Month, Count)
```

```
## # A tibble: 6 × 3
## # Groups:   Phenotype [2]
##   Phenotype Month Count
##   <chr>     <fct> <int>
## 1 FWR       Jan      26
## 2 FWR       Feb      22
## 3 FWR       Mar      12
## 4 MIG       Jan      17
## 5 MIG       Feb      24
## 6 MIG       Mar      20
```

```
mm %>% group_by(Phenotype) %>% summarize(Count=n())
```

```
## # A tibble: 2 × 2
##   Phenotype Count
##   <chr>     <int>
## 1 FWR          60
## 2 MIG          61
```

```
ggplot(mm, aes(x = `Fork Length`, fill=Phenotype)) +
  geom_density(alpha = 0.5) +
  facet_grid(Phenotype~Month) +
  theme_bw() +
  theme(panel.grid = element_blank()) +
  ylab("Density\n") +
  xlab("\nFork Length") +
  scale_fill_manual(values=c("red","blue"))
```

Testing for differences.

```
tester<-function(month, dataframe) {

  MIG<-dataframe %>% filter(Month== month, Phenotype == "MIG")
  FWR<-dataframe %>% filter(Month== month, Phenotype == "FWR")

  sx<-sd(MIG$`Fork Length`)
  sy<-sd(FWR$`Fork Length`)

ztest<-z.test(MIG$`Fork Length`, FWR$`Fork Length`, sigma.x=sx, sigma.y=sy)
return(ztest)

}
```

```
tester("Jan", mm)
```

```
## 
##  Two-sample z-Test
## 
## data:  MIG$`Fork Length` and FWR$`Fork Length`
## z = 5.4108, p-value = 6.275e-08
## alternative hypothesis: true difference in means is not equal to 0
## 95 percent confidence interval:
##  3.285512 7.017655
## sample estimates:
## mean of x mean of y 
##  70.88235  65.73077
```

```
tester("Feb", mm)
```

```
## 
##  Two-sample z-Test
## 
## data:  MIG$`Fork Length` and FWR$`Fork Length`
## z = 5.2736, p-value = 1.338e-07
## alternative hypothesis: true difference in means is not equal to 0
## 95 percent confidence interval:
##  4.227055 9.227490
## sample estimates:
## mean of x mean of y 
##  72.00000  65.27273
```

```
tester("Mar", mm)
```

```
## 
##  Two-sample z-Test
## 
## data:  MIG$`Fork Length` and FWR$`Fork Length`
## z = 4.9275, p-value = 8.331e-07
## alternative hypothesis: true difference in means is not equal to 0
## 95 percent confidence interval:
##  4.024946 9.341721
## sample estimates:
## mean of x mean of y 
##  73.35000  66.66667
```

Counts of sex:  
Sex 1=male, 2=female

```
meta %>% group_by(aggregated_new_classes, sex) %>% summarize(Count=n())
```

```
## # A tibble: 4 × 3
## # Groups:   aggregated_new_classes [2]
##   aggregated_new_classes   sex Count
##   <chr>                  <dbl> <int>
## 1 FWR                        1    34
## 2 FWR                        2    26
## 3 MIG                        1    32
## 4 MIG                        2    29
```

## V Misclassified Inds from KNN

Figure 4B, 7 individuals not correctly assigned.

```
knnresults<-read_csv("outputs/appendix-1/knn-results.csv")
```

```
total<-bind_cols(meta, knnresults) %>% filter(Otolith != Prediction) %>% filter(aggregated_new_classes!="MIG") %>%
  select(Sample_ID, aggregated_new_classes, new_classes, station, sex, SampleDate, lat, lon, station) %>%
  mutate(Phenotype="Misclassified FWR") %>% group_by(Phenotype, lon,lat) %>%
  summarize(Count=n())

total
```

```
## # A tibble: 1 × 4
## # Groups:   Phenotype, lon [1]
##   Phenotype           lon   lat Count
##   <chr>             <dbl> <dbl> <int>
## 1 Misclassified FWR -122.  38.3     7
```

```
total2<-bind_cols(meta, knnresults) %>% filter(Otolith != Prediction) %>% filter(aggregated_new_classes!="FWR") %>%
  select(Sample_ID, aggregated_new_classes, new_classes, station, sex, SampleDate, lat, lon, station) %>%
  mutate(Phenotype="Misclassified MIG") %>% group_by(Phenotype, lon,lat) %>%
  summarize(Count=n())

total2
```

```
## # A tibble: 1 × 4
## # Groups:   Phenotype, lon [1]
##   Phenotype           lon   lat Count
##   <chr>             <dbl> <dbl> <int>
## 1 Misclassified MIG -122.  38.3     1
```

```
top2013<-read_csv("metadata/2012-samples.csv")

plot2<-top2013  %>% mutate(Phenotype=ifelse(new_classes=="FWR1","FWR","MIG")) %>%
  group_by(Phenotype, lon, lat) %>%
   summarize(Count=n()) %>% unique() %>% bind_rows(total,total2)
```

```
mapbox <- c(-122.5, 37.75, -121.25, 38.5)
sfemap <- get_stamenmap(mapbox, maptype = "terrain-background")
basemap<-ggmap(sfemap) + 
  xlab("\nLongitude") +
  ylab("Latitude\n")
```

```
  basemap +
  geom_point(data=plot2, aes(x=lon, y=lat, fill=Phenotype, size=Count), alpha=0.75, pch=21) +
 # geom_label_repel(data=toplot, aes(x=lon, y=lat, label=paste0(new_classes, "-", Count))) +
  facet_wrap(.~Phenotype, ncol=2) +
  xlab("\nLongitude") +
  ylab("Latitude\n") +
  theme(axis.text.x = element_text(angle=45, vjust = 1, hjust=1)) +
#  theme(axis.ticks = element_blank()) +
  scale_fill_manual(values=c("red","blue","grey50","grey50")) +
  theme(panel.margin = unit(2, "lines")) +
  theme(strip.text.x = element_text(face="bold", size=12))
```

```
ggsave("outputs/appendix-1/classifications.jpeg")
```
